# Reference Interval for Glycated Albumin, 1,5-AG/GA, and GA/HbA1c Ratios and Cut-Off Values for Type 1, Type 2, and Gestational Diabetes: A Cross-Sectional Study

**DOI:** 10.3390/biomedicines12122651

**Published:** 2024-11-21

**Authors:** Yusra Al-Lahham, Waldemar Volanski, Liana Signorini, Ademir Luiz do Prado, Glaucio Valdameri, Vivian Rotuno Moure, Marciane Welter, Alexessander C. Alves, Marcel Henrique Marcondes Sari, Fabiane Gomes de Moraes Rego, Geraldo Picheth

**Affiliations:** 1 Graduate Program in Pharmaceutical Sciences, Federal University of Paraná, Curitiba 80210-170, Brazil; volanski@ufpr.br (W.V.); lianasig@ufpr.br (L.S.); gvaldameri@ufpr.br (G.V.); vivian.moure@ufpr.br (V.R.M.); marcelsari@ufpr.br (M.H.M.S.); rego@ufpr.br (F.G.d.M.R.); 2 Laboratory Division, Curitiba City Hall, Curitiba 80530-908, Brazil; 3 Federal Institute of Parana, Colombo 83403-515, Brazil; 4 School of Human Development and Health, Faculty of Medicine, University of Southampton, Southampton SO17 1BJ, UK; a.couto-alves@soton.ac.uk

**Keywords:** glycated albumin, 1,5-anhydroglucitol, glycated hemoglobin, diabetes, glycemic control, glycemic biomarkers, diabetes

## Abstract

**Background/Objectives:** Glycated albumin (GA) serves as a biomarker for short-term glycemic control (2–3 weeks), playing a role in diabetes management. Our goal was to establish reference intervals (RIs) for serum GA, and the ratios of 1,5-anhydroglucitol to GA (AGI) and GA to HbA1c in a Euro-Brazilian pediatric population (10 y, n = 299), adults (43.5 y; n = 290), and pregnant women (26 y, n = 406; 26.5 ± 3.1 gestation weeks). **Methods:** Receiver operating characteristic curve analysis was employed to determine RIs for type 1 diabetes (T1D) in children (n = 148) and adults (n = 81), type 2 diabetes (T2D, n = 283), and gestational diabetes mellitus (GDM, n = 177). **Results:** Both non-pregnant and pregnant women exhibited GA RIs of 10.0–13.3% and 10.6–14.7%, respectively. The AGI ratio varied from 1.2–4.3 in children, 0.9–3.6 in adults, and 0.8–3.1 in pregnant women. Meanwhile, the GA/HbA1c ratio ranged from 1.8–2.6 in children and adults to 2.3–3.6 in pregnant women. GA and AGI ratios accurately differentiated between T1D and T2D, demonstrating high sensitivity (>84%) and specificity (>97%), with AGI showing superior performance (AUC > 0.99). The GA/HbA1c ratio exhibited moderate discriminatory power (AUC > 0.733) but was less effective in distinguishing adult-onset T1D and T2D, suggesting its limited utility in certain groups. **Conclusions:** The proposed RIs are consistent with those of other Caucasian populations, affirming their relevance for Euro-Brazilian patients. The GA and AGI ratios emerge as valuable diagnostic tools for T1D and T2D, though their reduced sensitivity in diagnosing GDM warrants further investigation. Clinicians might leverage GA and AGI ratios for more tailored diabetes management, especially when HbA1c results are not optimal.

## 1. Introduction

Diabetes mellitus is a pathology that affects approximately 10% of the global population, with an increasing prevalence in developing countries [1]. The defining characteristic of diabetes is chronic hyperglycemia, which is a crucial element in the diagnosis and monitoring (control) of the pathological process [2,3,4]. It is imperative to control diabetes with insulin or oral hypoglycemic therapies to ensure the life of people with diabetes while simultaneously minimizing the progression of diabetic complications, promoting increased life expectancy and quality of life [5].

Among the laboratory biomarkers for the diagnosis and control of diabetes, the following have been established and consolidated: glycemia (fasting or random) and glycated hemoglobin A1c fraction (HbA1c), the latter considered the gold standard [6,7,8]. Other glycemic status biomarkers, such as 1,5-anhydroglucitol (1,5-AG) and glycated albumin (GA), have also been long recognized and are recommended with caution [9,10,11,12,13].

The 1,5-AG, a naturally occurring dietary polyol, is a marker of postprandial hyperglycemia, exhibiting sensitivity to rapid fluctuations in blood glucose levels or glycemic excursions within a few days [14]. Increased blood glucose levels are associated with reduced serum 1,5-AG concentrations. The biomarker reflects the glycemic status over 48 h to 2 weeks before the assay [15,16].

Glycated albumin, a ketamine, characterizes a non-enzymatic interaction (glycation) between serum glucose and albumin and reflects the average blood glucose concentration in a period of 2–3 weeks before the test, resulting from the half-life of serum albumin [17,18]. Capturing average blood glucose in a significantly shorter time compared to HbA1c (average blood glucose from the past 2–3 months), GA is a biomarker that identifies glycemic changes more quickly and efficiently [19,20,21]. It has been suggested that GA may serve as an indicator of glycemic control better than HbA1c in patients undergoing dialysis, as well as those presenting with retinopathy, nephropathy, and cardiovascular complications associated with diabetes [22,23,24]. Comparisons between the main characteristics of the biomarkers involved in this study are presented in Table 1.

New diagnostic parameters using GA in association with other biomarkers have been proposed, more notably 1,5-AG/GA ratio as the AGI index and GA/HbA1c ratios. These ratios aim to identify various characteristics of biomarkers in relation to DM, providing approaches for differential diagnosis. For instance, in cases of hyperglycemia, 1,5-AG concentrations in the blood decrease, while GA levels increase, leading to a higher AGI ratio. This provides an opportunity for more precise differentiation of DM or its associated factors. The GA/HbA1c ratio involves markers that capture average blood sugar levels over different periods, thereby allowing for more detailed monitoring of glycemic variations.

1,5-AG/GA (AGI) has facilitated the differential diagnosis of fulminant type 1 diabetes (FT1D) [34]. The GA/HbA1c ratio, with more reports in the literature, has been suggested to aid in predicting complications in the children of mothers with gestational diabetes (GDM) [35], with BMI in type 2 diabetes (T2D) [36], and in type 1 diabetes (T1D) individuals with autoantibodies against insulin with high binding capacity and low affinity or recognition of diabetic nephropathy in T2D [37,38].

Considering the increasing possibilities of the application of GA in diabetes, we propose to extend the knowledge of this biomarker, determining the reference interval as well as the ratios of GA with 1,5-AG and HbA1c, by studying these parameters in a southern Brazilian population, including data from children. We also evaluated these parameters’ diagnostic criteria (cut-off values) for T1D in children and adults, T2D, and GDM.

This cross-sectional study introduces further diagnostic and monitoring tools for DM, contributing to the essential ongoing expansion of knowledge regarding this condition. A STROBE Statement—Checklist for cross-sectional studies is presented in Supplementary Materials Table S5.

## 2. Materials and Methods

### 2.1. Subjects and People with Diabetes

These individuals were classified as children (n = 299; 6–14 years), adults (n = 290; 21–62 years), and pregnant women (n = 406, 14–44 years). Individuals in the control groups had no pre-existing chronic diseases or other health conditions, including, for pregnant women, diabetes before pregnancy. The groups were selected from schools (children), blood banks (adults), and the Pregnancy Care Center at the Clinical Hospital of the Federal University of Paraná; the participants are residents of Curitiba (Paraná State, southern Brazil), which has an admixture population, phenotypically characterized by 85% Euro-Brazilians, 14% Afro-Brazilians, and 1% Asian and Indigenous. This study was approved by the Research Ethics Committee under CAAE nos. 24676613.6.0000.0102, 68027317.7.0000.0102, and 05335612.4.0000.0102.

For comparisons with healthy groups, patients with early- (children, n = 148) and late-onset T1D (adults, n = 81), T2D (n = 283) and pregnant women with GDM (n = 177) were selected. Pregnant women under 18 years of age in the control group represent approximately 3% of the total sample. Diabetes was established using criteria from the American Diabetes Association [39]. Briefly, blood glucose ≥ 126 mg/dL and/or HbA1c ≥ 6.5% (48 mmol/mol) for T1D and T2D, and fasting oral glucose tolerance test (75 g) ≥ 92 mg/dL or 1 h ≥ 180 mg/dL or 2 h ≥ 153 mg/dL for GDM.

### 2.2. Inclusion/Exclusion Criteria

For individuals characterized as healthy (controls) by the absence of clinical manifestations or use of any medication for diabetes, thyroid or hypotensive, and laboratory parameters for renal function (creatinine < 1.2 mg/dL), liver function (aspartate aminotransferase [AST] < 40 U/L and alanine transaminase [ALT] < 45 U/L), and glycemia (glycemia < 99 mg/dL and/or HbA1c < 5.7%) were selected. The exclusion criteria were albumin < 3.0 g/dL or the presence of anemia (hemoglobin < 7 g/dL).

People with T1D and GDM were treated with insulin (regular and NPH). In T2D, individuals were treated with metformin hydrochloride or glibenclamide (sulfonylurea), as deemed appropriate. People with T2D did not report using SGLT2 inhibitors (gliflozins) or alpha-glucosidase inhibitors (acarbose), as observed in the medical records. People with diabetes with proteinuria and overt nephropathy (creatinine > 1.5 mg/dL), liver disease (AST and ALT > 45 U/L), or in the case of T1D, presenting symptoms of ketoacidosis, were excluded.

### 2.3. Biomarker Measurements

Blood glucose, collected in a NaF-EDTA tube (BD Vacutainer^®^, Franklin Lakes, NJ, USA), was quantified in fasting conditions (pregnant women and T2D) and randomly (T1D) using the hexokinase-UV method. The other laboratory parameters were quantified in serum (SST^®^ II Advance^®^, BD Vacutainer^®^, Franklin Lakes, NJ, USA) and were performed using routine automated methods. The samples were stored at −80 °C until analysis. Samples at −70 °C are suitable for GA for up to 23 years [26].

Then, HbA1c was quantified in whole blood (EDTA K_2_; BD Vacutainer^®^, Franklin Lakes, NJ, USA) by immunoturbidimetry (HbA1c Turbiquest, Labtest, Brazil), followed by 1,5-AG quantified enzymatically using the GlycoMark reagent (GlycoMark, Raleigh, NC, USA). Glycated albumin was quantified with the enzymatic reagent (Lucia GA-L, Asahi Kasei Pharma, Shizuoka, Japan), with results expressed as a percentage (%). The conversion of the GA reported in percentage (%) to mmol/mol can be performed with the conversion equation GA (%) = 0.05652 × GA (mmol/mol) − 0.4217, proposed by the literature [6]. All analyses were performed using an automated system (LabMax 400, Labtest, Brazil).

The 1,5-AG/GA ratio or AGI (µg/mL%) was calculated with 1,5-AG in µg/mL and GA in %. We chose not to express the 1,5-AG/GA ratio units to facilitate text reading. The GA/HbA1c ratio was obtained with GA in % and HbA1c in %; therefore, it does not present unity.

### 2.4. Statistics Analysis

For continuous variables, normality was verified using the Kolmogorov–Smirnov test. Variables with normal distribution were presented as mean ± standard deviation and compared using the independent two-tailed Student’s *t*-test. In the absence of normal distribution, results were presented as median (interquartile range, 25–75%) and compared using the Mann–Whitney U test. Qualitative variables were presented as “n” or percentages and compared using the chi-squared test.

The reference interval was calculated according to CLSI C28-A3, based on the normal distribution method (mean ± 2-SD) and the non-parametric percentile method (2.5–97.5%), as appropriate using the MedCalc program. Outliers were identified using Tukey’s test with MedCalc (Q1 − 1.5 × IQR and Q3 + 1.5 × IQR), where Q1 = quartile 1, or 25%, and Q3 = quartile 3 or 75%, and IQR = interquartile range) and removed once.

Receiver operating characteristic (ROC) curves and associated parameters (sensitivity, specificity, cut-off criteria) were performed using the MedCalc program. Additionally, the MedCalc program (version 22.023, MedCalc Software, Ostend, Belgium), Statistica (version 14, Cloud Software Group, Tulsa, OK, USA), Data Science Workbench (version 14), and GraphPad Prism (version 10.0.0) were used in the analyses. A probability of less than 5% (*p* < 0.05) was considered significant in all comparisons.

## 3. Results

The characteristics of the control groups are presented in Table 2.

The anthropometric and laboratory parameters of the control group are within the reference interval expected for a healthy population when adjusted for age and pregnancy [40]. As expected, the significant difference observed in the comparisons is mainly due to the presence of pregnancy, which is known to alter serum biomarkers.

The minimum and maximum concentrations for non-fasting blood glucose and HbA1c were 55–99 mg/dL and 4.0–5.7% for the children, 56–99 mg/dL and 4.3–5.7% for adults, and 59–91 mg/dL and 3.5–5.5% for pregnant women.

Notably, the related individuals did not present laboratory alterations with predictable effects on the analyses of the biomarkers, justifying the consistency of the groups chosen for the present work.

### 3.1. Reference Interval for Glycated Albumin

The reference interval for GA with the enzymatic method is presented in Table 3.

The MedCalc program employed uses the CLSI C28-A3 recommendations to calculate the reference interval. Considering the sample size of the groups and the normal distribution verified by the Kolmogorov–Smirnov test, the results are presented for the method based on the normal distribution (mean ± 2 standard deviations), with 95% of the reference interval (two-sided).

The mean concentration of GA for the children (11.8 ± 0.89%) and adults (11.9 ± 1.02%) groups did not differ (*p* = 0.205), suggesting that the use of the reference interval for 6 to 62 years (non-pregnant individuals) of 10.0% to 13.8% can be used in this population.

**Figure 1:**
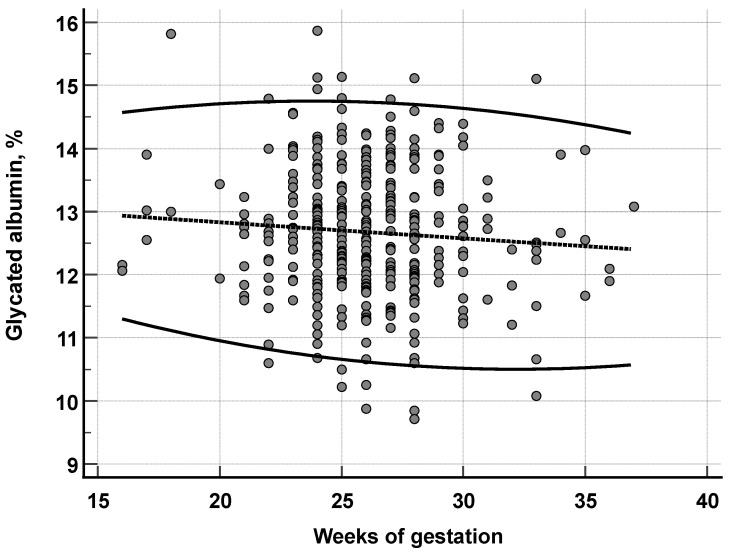
Reference interval for glycated albumin during pregnancy related to the weeks of gestation. The dotted line represents the median and solid lines represent the 0.25 and 0.975 percentiles.

The GA concentrations in the presence of pregnancy (16–37 weeks) were, on average, 5.8% higher than in the adult group (11.9 vs. 12.6%; *p* < 0.001) and 6.6% higher than the child group (11.8 vs. 12.6%; *p* < 0.001). The proposed reference interval for GA was 10.6 to 14.7% in pregnant women; therefore, higher than the other groups. The evolution of GA concentrations during pregnancy is shown in Figure 1.

The GA concentration during pregnancy in the study population showed, on average, a slow reduction from 12.9% at the 16th week of gestation to 12.4% at the 37th week. This variation of 0.5 in the GA concentration throughout pregnancy is discreet, and the observed reference interval of 10.6–14.7% can be suggested for the entire gestational period in terms of diagnostic use.

### 3.2. Reference Interval for 1,5-Anhydroglucitol to Glycated Albumin Ratio

The reference range for the study groups for the 1,5-AG/GA ratio (AGI) is presented in Table 4.

Comparing the mean AGI between children and adults, 2.45 ± 0.75 vs. 1.91 ± 0.64 (*p* < 0.001) and adults and pregnant women, 1.91 ± 0.64 vs. 1.56 ± 0.59 (*p* < 0.001), the 1,5–AG/GA (AGI) ratio significantly differs between the groups.

With these characteristics, the reference interval for AGI for children 1.2–4.3, adults 0.9–3.6, and pregnant women 0.8–3.1 should be applied.

### 3.3. Reference Interval for Glycated Albumin to Glycated Hemoglobin (GA/HbA1c)

The reference interval for the GA/HbA1c ratio is presented in Table 5.

No significant differences between children and adults were observed in the mean GA/HbA1c ratios, 2.27 ± 0.21 vs. 2.25 ± 0.23 (*p* = 0.136). Conversely, the comparison between adults and pregnant women was 2.25 ± 0.23 vs. 2.81 ± 0.34, which were significantly different (*p* < 0.001). With these characteristics, the reference interval for the GA/HbA1c ratio for the study population can be characterized as 1.8–2.6 (non-pregnant individuals). Pregnant women did not present a normal distribution and showed a large amplitude of RI (3.64–2.32; RI 1.33) compared to non-pregnant individuals.

### 3.4. Effect of Sex on the Reference Interval in the Biomarkers

Comparisons between men and women associated with GA, 1,5-AG/GA, and GA/HbA1c concentrations are presented in the Supplementary Materials (Tables S2–S4). There were no significant differences between the groups regarding GA and GA/HbA1c ratio in the people with diabetes and the presence of diabetes. However, the 1,5-AG/GA (AGI) ratio was higher in the male sex within the child and adult controls (*p* < 0.05), with no differences observed in the groups of people with diabetes.

### 3.5. People with Diabetes—Group Characteristics

We established patients into four groups to determine cut-off values and sensitivity characteristics of the biomarkers for diabetes. The primary characteristics of these groups are presented in Table 6 and expanded form in Table S1 (Supplementary Materials).

The selected T1D (children and adults) and T2D diabetic groups were diagnosed with well-established, long-term criteria from the American Diabetes Association [39], and both groups presented inadequate glycemic control (HbA1c target <7.0%). Moreover, the GDM group met the expected diagnosis criteria after excluding pregnant women with pre-gestational diabetes and diabetes during pregnancy, as per official guidelines [39]. The median age in the T1D group was similar to the control groups, while the T2D group was significantly older. Pregnant women with GDM also had a higher median age compared to the controls (31 vs. 26, *p* < 0.001). None of the groups showed abnormalities in renal function (creatinine > 1.5 mg/dL), nutrition (serum albumin > 3.5 g/dL, and for pregnant women > 2.8 g/dL), and liver function (AST and ALT < 45 U/L).

### 3.6. Cut-Off Values for Diabetes with Glycated Albumin, 1,5-AG/GA, and GA/HbA1c Ratios

**Figure 2:**
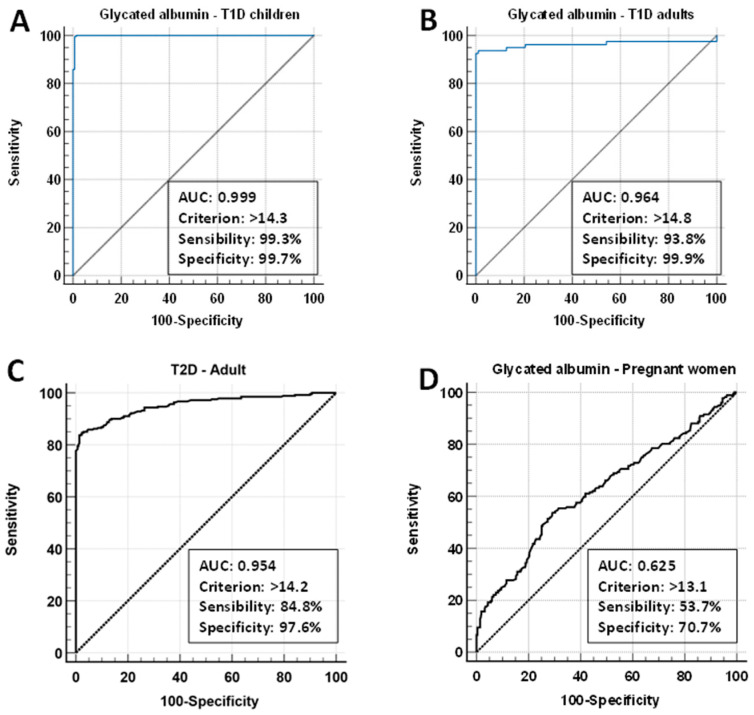
Receiver operating characteristic curve for glycated albumin and different types of diabetes. (**A**) T1D: type 1 diabetes (<14 years), (**B**) T1D: type 1 diabetes (37–53 years), (**C**) T2D: type 2 diabetes (41–66 years), and (**D**) pregnant women (24–35 years). AUC: area under curve; criterion: cut-off value.

Figure 2 presents the cut-off values and sensitivity and specificity values for GA across the different types of diabetes, determined using ROC curve analysis.

The sensitivity and specificity values parameters for GA are presented in Table 7.

**Figure 3:**
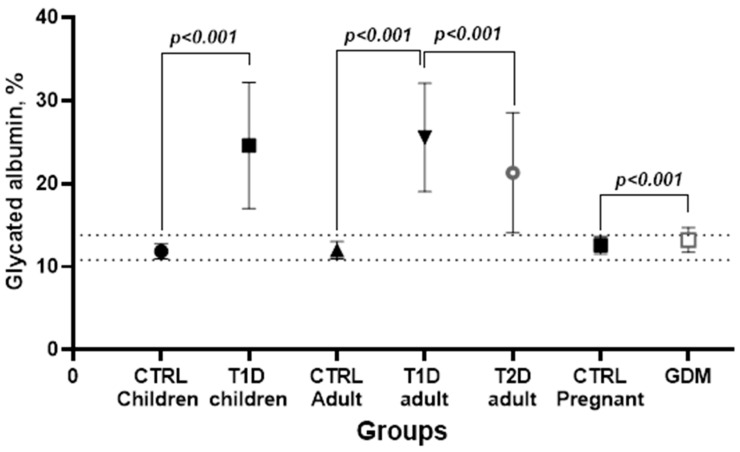
Comparisons of glycated albumin between healthy patients and different types of diabetes. The values are mean ± standard deviation, the sample size for each group is described in Materials and Methods. CTRL: control; T1D: type 1 diabetes; T2D: type 2 diabetes; GDM: gestational diabetes mellitus. The dotted lines represent the proposed reference interval for children and adults combined (10.0–13.8%), *p*: probability using the two-tailed Student’s *t*-test.

For the T1D (children and adults) and T2D groups, the ROC curve showed an AUC greater than 0.9, indicating excellent discrimination [41]. In these groups, the cut-off values for the GA ranged from 14.2 to 14.8%. In contrast, albumin demonstrated poor discrimination, with an AUC of 0.625 for GDM, sensitivity of 53%, and specificity of 70.7% for a cut-off value of 13.1%. The comparison of mean GA levels between controls and people with diabetes, highlighting the biomarker’s discriminatory ability within the reference interval, is presented in Figure 3.

The T1D groups (children and adults) showed the best discrimination with GA, considering the proposed reference interval (Table 3). The T2D group also demonstrated good discrimination, though the result dispersion (SD) approached the upper limit of the reference interval. The GDM group, however, was not efficiently discriminated against and remained indistinct when applying the general RI to GA.

### 3.7. Cut-Off Values for Diabetes with 1,5-AG/GA (AGI) and GA/HbA1c Ratios

Four patient groups with different types of diabetes were created to establish cut-off values and sensitivity characteristics of the biomarkers for diabetes (Table 8 and Table 9). The primary characteristics of these groups are outlined in Table 6, with further details provided in Table S1 (Supplementary Materials).

The AUC greater than 0.99 shows excellent discrimination for the 1,5-AG/GA ratio, with cut-off values below 0.79 for T1D children, 0.90 for T1D adults, and 1.0 for T2D, yielding sensitivity and specificity above 96%. For GDM, the AUC of 0.70 reflects moderate or regular discrimination, with a cut-off value below 1.3, providing 63% sensitivity and 72% specificity.

The GA/HbA1c ratio showed varied discrimination across the study groups. The best discrimination was observed in T1D children, with an AUC of 0.93 and sensitivity and specificity higher than 87% for a cut-off value above 2.5. In T1D adults, diabetes discrimination was also good (AUC 0.867), though sensitivity was lower (76%) compared to T1D children (87%), with a similar cut-off value (2.56). For T2D, discrimination was notably lower (AUC 0.733), with sensitivity reducing to 61%, with a cut-off value of 2.42.

The GA/HbA1c ratio moderately characterized the GDM group in a moderate way (AUC 0.677), with sensitivity and specificity both under 75%. Interestingly, the GDM group’s cut-off value was lower than that for the other groups, with ratios below 2.66 facilitating group discrimination. The mean GA concentration in the GDM group was 5.4% higher than in the control group (13.29 ± 1.53 vs. 12.6 ± 1.05%; *p* < 0.001), and the HbA1c concentration was 8.7% higher in the GDM group compared to the controls (5.0 ± 0.38 vs. 4.5 ± 0.37; *p* < 0.001).

## 4. Discussion

Glycated albumin captures the average blood glucose level between 2–3 weeks before the test [42] and is considered a short- to moderate-term biomarker compared to long-term glycated hemoglobin to access glycemic status [43]. The possibility of quantifying GA without fasting, its stability in serum [44], and the automated, reproducible enzymatic methods make this biomarker attractive for the clinical laboratory [45,46].

According to Timbrell [47], reference intervals (RIs) are a range of values supplied alongside laboratory measurements for comparison to allow interpretation of this data. Considering that biomarkers may present variations associated with the characteristics of different populations, the establishment of the RI constitutes an essential parameterization for use in diagnosing these markers.

### 4.1. Glycated Albumin Reference Interval for Pregnant and Non-Pregnant Individuals

Using enzymatically quantified GA (Lucica-GA-L), we established a RI for a mixed population from southern Brazil, predominantly Euro-Brazilians (85%). The interval for children (6–14 y) and adults (21–62 y) combined (n = 589) was found to be 10.0–13.8% (Table 3). When applying the equation proposed by Sato et al. [6]. for SI units, the corresponding values are 184–252 mmol/mol. Our study revealed no differences in GA concentrations between adults and children (Table 3) or between sexes (Table S2).

The literature has no consensus on whether GA presents differences between sexes, age, ethnicity, and body mass index. While some studies report statistical differences in these factors, they are often small and lack clinical relevance [48]. Comparisons with the reference interval from other populations using enzymatic methods are summarized in Table 10.

The RI for GA in the Brazilian population under study was similar to the American population (9.9–14.2%) and the Chinese population (10.4–13.9%). In contrast, Asian populations such as South Koreans and Japanese (except for neonates) and South Africans exhibit higher concentrations with greater amplitude (ΔRI) of GA than our findings. In these cases, the upper reference for GA was roughly 1–3.7% higher than that observed in our study (13.8%).

The RI for Italian populations highlights elements for discussion (Table 10). In a study by Bellia et al. [52], the upper limit for GA was established at 12.2% (n = 1334), while Paroni and peers [29] reported a limit of 16.9% (n = 32). The enzymatic methodologies in these cases were different and especially relevant, and the sample sizes (n) were disparate. Therefore, the establishment of the RI reflects the characteristics of the population, the methodological characteristics of the quantification, and the RI calculation. For small sample sizes (usually n < 120), it is recommended that the RI be calculated using the robust method (CLSI C28-A3), which provides a broader range compared to the method based on the normal distribution employed in our study.

Chume et al. [49] studied a Brazilian population from southern Brazil (82% Euro-Brazilians) and found a higher upper limit than our study (18.0% vs. 13.8%), along with the most significant variation (ΔRI 7.2); the population of our study presents ethnic similarities with those described by the researchers. Possible explanations for the differences in findings may be associated with the selection criteria for non-diabetic individuals, experimental design not being explicitly aimed at deriving the RI (as it was estimated from the mean ± standard deviation), the removal of outliers, and, primarily, the methodology employed. Chume et al. [49] used the GlycoGap reagent, which indicates an RI for GA of 11–16%, while the Lucica-GA-L method, the most widely used, minimizes this differential factor.

Finally, it is noteworthy that serum GA concentrations measured in healthy dogs, predominantly Labrador Retrievers, using the Lucica-GA-L method, yielded a median of 11.7% (95% CI, 11.4–12.0%), which aligns closely with human studies of Sako et al. [65].

In our Euro-Brazilian study population, we established the RI at 10.6–14.7% for 16–37 weeks of gestation (Table 3). The sample of pregnant women in the study does not allow stratification into the three trimesters of pregnancy, as is typical in other studies. Our sample does not include pregnant women in the first trimester (up to 13 weeks of gestation) and concentrates the gestational period between 24–28 weeks (>65%). This distribution is influenced by standard protocols for diagnosing GDM, which recommend this gestational period for risk assessment.

The attempt to capture the effect of GA in the gestational period revealed a slight decline in concentration as the pregnancy progressed (Figure 1). A GA behavior during pregnancy is comparable to that observed in our study, which was reported by Dong et al. [64] in a Chinese population (Table 10). As can be seen, other studies presented a similar pattern to the one observed in our study. An exception is the study by Pang et al. [63], using a longitudinal approach, which reported significantly higher GA concentrations (e.g., 38.4%) than other studies. The study was not deemed ineligible for consideration in the comparative processes due to its distinctive characteristics.

Our results are generally comparable to those observed in Caucasian and Chinese populations, while Japanese populations show RI with higher concentrations (Table 10). It is noteworthy that the variation in the reference interval (maximum–minimum) does not present relevant differences (ΔIR 3.5–5.6) across the studies, excluding the study by Pang et al. [63].

Araki et al. [56] evaluated GA in 3.14 million Japanese blood donors, presenting an increase with age, exemplified by 13.5 ± 1.0% for the 16–19 year and 14.8 ± 2.4% for the 60–69 year (Table 10). They also reported a significantly higher GA concentration in women than men, a trend not seen in our study. For the entire study population (n = 3,142,794) aged 16–69 years, the mean GA was 13.8 ± 1.7%, with an estimated RI (mean ± 2SD) of 10.4–17.2%. Comparatively, our lower RI limit (10.0%) is similar, but the upper limit (13.8%) is about 20% lower, possibly attributed to the ethnic differences between the populations.

Sugawara and coworkers [66] examined GA levels in mothers with diabetes and their children (n = 77). They found higher levels of GA in mothers whose newborns had complications such as neonatal hypoglycemia, respiratory disorders, hypoglycemia, myocardial hypertrophy, and large-for-date status. The authors concluded that GA is useful for predicting pregnancy outcomes in mothers with GDM. Their cut-off values (13.6–14.7%) are consistent with those observed in our study.

### 4.2. GA and Diabetes Cut-Off Values for Pregnant Women

In the absence of pregnancy, we noted that GA discriminated T1D (children and adults) and T2D with high sensitivity and specificity in patients with well-established diagnoses (Figure 2). The observed cut-off values of 14.2–14.8% are close to the upper limit of the reference interval (13.8%).

Shimizu et al. [67] and Freitas et al. [44] analyzed studies in Asian populations (Japanese, Chinese, and South Koreans) and found varying cut-off values. For patients with established diabetes, the cut-offs reached 17%, while for recently diagnosed individuals, they ranged between 14.7 and 15.7%. Our data align with those for recent diagnoses, and the sensitivity and specificity of only two studies mentioned in Japanese and South Koreans were above 83% in these groups.

In a meta-analysis covering nine studies, mainly comprising Orientals and more than 10,000 individuals, Chume et al. [49] identified cut-off values for diabetes of 15.0 and 17.1% [68]. These authors indicate the optimal cut-off value of 17.1%, about accuracy, with sensitivity of 55.1% and specificity of 94.4%, suggesting this is optimal for diagnosing diabetes in previously undiagnosed individuals. Chume et al. [69] also showed that in Brazilian COVID-19 patients, GA could diagnose new diabetes with a cut-off value of 19%, showing high specificity (85%) but low sensitivity (48.4%).

Dozio et al. [70], examining Araki et al. [55] with more than 3 million Japanese patients, described GA in healthy individuals < 15.6% and blood donors with GA ≥ 16.5 being at higher diabetes risk. Given that the Japanese population upper GA limit (17.2%) is about 20% higher than that in our Brazilian cohort, the upper limit that we describe for adults and children (13.8%) corrected by this factor (16.5%) agrees with the proposed concentration presented by these authors. Certainly, studies with larger sample sizes will be necessary to confirm this estimate.

The GA’s ability to discriminate GDM in our population was limited (Table 3, Figure 2). The ROC curve identified a cut-off value of 13.1%, overlapping with the RI for this population (10.6–14.7%).

Our results were similar to those described by Dong et al. [64], who showed that GA is inadequate for the diagnosis of GDM (AUC 0.503) and for predicting adverse pregnancy outcomes in a Chinese population. Additionally, Pang et al. [63] evaluated a population of multiracial North American pregnant women in a longitudinal study and showed that plasma GA was not a sensitive marker of glycemic metabolism. In the study, GA did not correlate with glucose, insulin, or even HbA1c concentrations, concluding that this biomarker does not appear suitable during pregnancy.

As a counterpoint, applying logistic regression, Sugawara et al. [71], evaluating Japanese mothers with GDM (n = 112), showed that GA in the third trimester with a cut-off value of 13.5% (AUC 0.797; sensitivity 90.4% and specificity 64%) can predict infant complications, reinforcing the usefulness of this biomarker. Therefore, the possibility of new studies of GA in the characterization of diabetes during pregnancy and the confirmation of pregestational diabetes in groups that require new markers remains open.

The differences between the cut-off values were expected, as criteria for establishing diabetes (retinopathy, oral glucose tolerance test, fasting glycemia, HbA1c) vary with the specificities of the population under study, which can capture diabetes in more or less its early stages, affecting the response of biomarkers, particularly GA.

Our study addresses different types of diabetes, characterized by parameters established in international guidelines, allowing a differentiated detailing of the biomarkers evaluated. The 1,5-AG/GA ratio or AGI captures the antagonistic effect of two biomarkers in response to increased glycemia. Hyperglycemia favors the increase in glycation and serum GA while simultaneously promoting a reduction in 1,5-anhydroglucitol concentration [34].

For the study population, the AGI reference interval differs for children (1.2–4.3), adults (0.9–3.6), and pregnant women (0.8–3.1), as shown in Table 4 and Table S3. Differences in the RI for AGI were expected because 1,5-AG presents variations between sexes and ages, as described by Welter et al. [27]. ROC curve analyses show that cut-off values for AGI in T1D children (≤0.797), T1D adults (≤0.901), and T2D (≤1.08) in the study are discriminated with proven efficacy by sensitivity and specificity greater than 95% (Table 7). A cut-off value for AGI ≤ 1.39 inefficiently discriminates GDM due to sensitivity or specificity below 75% (Table 7). We hypothesize that the moderate increase in glycemia over a short period (months), characteristic of GDM, does not allow significant changes in the biomarkers that make up the AGI, explaining the low performance of this ratio in discrimination.

In a population of Chinese adults (median 36 years) without diabetes, a median AGI of 1.57 (1.22–1.98, interquartile range) was observed by Ying and collaborators [34], similar to our study. In their study with a small sample size, the authors reported for fulminant T1D (FT1D; n = 20), autoimmune T1D (T1DA; n = 47), latent autoimmune diabetes in adults (LADA, n = 36), and T2D (n = 42) for AGI, respectively (median, 25–75%), as 0.16 (0.10–0.25), 0.21 (0.11–0.41), 0.18 (0.09–0.94), and 0.62 (0.21–0.94).

By applying the RI proposed in our study (Table 4), only T2D would be expected to have some overlap with healthy individuals, and the other types of diabetes primarily associated with insulin insufficiency (FT1D, T1DA, and LADA) would be distinguishable. The proposed cut-off values for AGI would also allow for effective discrimination of these groups. The AGI was proposed to discriminate FT1D, a subgroup of idiopathic T1D, characterized by rapid processes of destruction of pancreatic beta cells, progression of hyperglycemia, and ketoacidosis [34,72]. According to Imagawa et al. [73], who first described FT1D, it occurs in approximately 20% of T1D with ketosis-onset T1D.

In this condition of rapidly evolving hyperglycemia, HbA1c may not adequately capture this elevation, and biomarkers that identify early glycemic changes, such as 1,5-AG (1–2 weeks) and GA (2–3 weeks), allow one to discriminate FT1D. We believe that our study expands knowledge of the characteristics of AGI as a biomarker in diabetes and may contribute to its rational use in the diagnosis of the FT1D subtype, as well as possibly other specific subtypes of diabetes.

The relationship between AGI and the different types and subtypes of diabetes and their complications is rarely explored in the literature. AGI requires a more significant number of robust studies that explore the characteristics of the biomarkers involved before they can be incorporated into clinical practice.

### 4.3. Glycated Albumin/Glycated Hemoglobin Ratio for Non-Pregnant Women

The GA/HbA1c ratio is composed of two biomarkers that increase in the presence of hyperglycemia. Our study established the GA/HbA1c ratio and RI for adults and children at 1.8–2.6, with no differences identified between sex and age (Table 5 and Table S4). GA/HbA1c ratio has been described in South Korean adults (49 years), with standard glycemic curve and pre-diabetics, as 2.06 ± 0.28 [74], and Japanese adults (64 years) at 2.55 ± 0.53 [38]; these values are similar to those of our study (Table 5). For Japanese children (12.3 years), a ratio of 2.20 ± 0.24 has been reported, which are values at the upper limit of the reference in our study for children (1.8–2.6) [75].

Kim et al. [76] evaluated patients with T2D and showed that the GA/HbA1c ratio significantly correlates with insulin secretory function but not insulin resistance. With a cut-off value of 2.71 for the GA/HbA1c ratio, Wang et al. [38] obtained the prediction of diabetic nephropathy in patients with T2D with sensitivity and specificity, respectively, 67.7 and 77.8%. The proposed cut-off value is higher than what we identified for T2D (>2.42), and this may be due to our group not presenting signs of nephropathy.

In a study with a small sample size (n = 48) [37], it was identified that the GA/HbA1c ratio is higher in patients who have insulin antibodies (InsAb) with high binding capacity and low-affinity properties compared to those patients with negative InsAb with T1D (3.78 ± 0.63 vs. 2.99 ± 0.22; *p* = 0.001) and T2D (3.36 ± 0.79 vs. 2.61 ± 0.48; *p* = 0.003). InsAb with high binding capacity and low affinity is rare, albeit identifying this group of patients is relevant because these antibodies can bind with a large amount of insulin and rapid release of this hormone, significantly affecting glycemic control, including episodes of morning hypoglycemia.

During pregnancy, our study showed a reference interval for 16–37 weeks of gestation for GA/HbA1c of 2.3–3.6 higher and with greater amplitude when compared to the absence of pregnancy (Table 5). Our study captures GA/HbA1c ratio, particularly between 24–28 weeks of gestation (6–7 months).

Sugawara et al. [35] studied 77 Japanese pregnant women with GDM, classified by the presence of complications in the newborns (shoulder dystocia, hypoglycemia, myocardial hypertrophy, respiratory disorders, hypocalcemia, hyperbilirubinemia, newborns being large for gestational age and polycythemia), and those without complications. GA/HbA1c ratio was able to predict (ROC curve analysis, sensitivity/specificity) myocardial hypertrophy (AUC 0.91; 96%/81%) and large size for gestational age (AUC 0.86; 92%/76%) with a cut-off value of 2.55. Notably, GA alone was also capable of similar discrimination with cut-off values of 13.9 and 14.1% for these same patients, a higher cut-off value than that presented for our GDM group (GA > 13.1) where these complications were not evaluated (Figure 2).

The GA/HbA1c ratio showed a different characteristic in our study for GDM (Table 8). The cut-off value established by the ROC curve showed a signal in the opposite direction compared to the other types of diabetes studied. For GDM, a cut-off value lower than 2.6 (≤2.66) discriminates GDM with sensitivity and specificity of 62 and 68%, respectively, with AUC 0.677, considered low to moderate.

The GDM and control groups under study had similar gestational ages (25.4 ± 3.7 vs. 26.5 ± 3.1 weeks of gestation), with the GDM having a significantly higher age and BMI than the control group (Table 2 and Table S1). We hypothesize that the effect observed in the study may be due to some uncontrolled factors. Microalbuminuria or mild albuminuria (not verified), common factors during pregnancy, may be present. This loss of albumin associated with a higher HbA1c in the GDM group may provide the effect of reducing the GA/HbA1c ratio.

We do not rule out the possibility that this finding is coincidental, resulting from inadequate pairing or other factors that may influence the GA/HbA1c ratio, which was not controlled in the groups with pregnant women. Further studies will be necessary to evaluate this finding. We did not identify a similar published study to compare our results.

Koga et al. [77] presented disease conditions compatible with low and high GA/HbA1c ratios. From this study, among the various diseases and conditions described, we highlight that a deterioration in glycemic control and neonatal diabetes is associated with high ratios of this biomarker and the rapid improvement in glycemic control, iron deficiency anemia, and pregnancy with a reduction in this ratio. The AG/HbA1c ratio has the potential to be a marker of interest for diagnosis and glycemic control in diabetes, requiring a more significant number of studies to substantiate this hypothesis.

## 5. Study Limitations

This study has some limitations. The complications of diabetes or people with diabetes with recent diagnoses were not analyzed to assess the performance of the biomarkers. In the people with diabetes groups, some effects, such as microalbuminuria, hyperthyroidism, and others that may affect the results of GA, were not excluded. In particular, in the analyses of pregnant women, the performance of GA was not analyzed, with calculation power, throughout the gestational period, leaving a gap for the first trimester.

Nevertheless, our study presents a relevant sample size for the reference interval for all groups, which provides consistency to the data presented. Information on the RI for the AGI and GA/Hb1c ratios is scarce in the literature, and we introduced this information in a structured and consistent manner.

Another characteristic that can enhance GA and its use as a biomarker in non-invasive samples is its unique diagnostic potential, as demonstrated by Aihara et al. [78]. Their study highlighted that GA quantification via LC-MS/MS shows a robust correlation with enzymatic measurement in blood (Lucica-GA-L) and extends this relationship to non-invasive samples such as tears (r = 0.793) and saliva (r = 0.927) with significant *p*-values (*p* < 0.001). These findings suggest that GA, when detected in alternative, non-invasive samples, could offer valuable diagnostic insight, reducing patient discomfort and healthcare burden. This preliminary evidence opens up new avenues for diabetes management, promoting more accessible diagnostic approaches.

In this research, the performance of GA and the ratios under study against different types of diabetes, in particular the T1D children group, offers consistent information for future applications in diabetes. Different forms of diabetes, such as maturity-onset diabetes of the young (MODY), stages of risk for diabetes (prediabetes), and complications of diabetes, must be evaluated with new studies and adequate experimental design using the promising biomarkers under study. This pathology demands new and efficient biomarkers for diagnosing and monitoring glycemia.

## 6. Conclusions

The reference interval for GA quantified by enzymatic assay in the Euro-Brazilian population shows no difference between sexes and ages, characterized as 10.0–13.8%. During pregnancy (16–37 weeks), concentrations of 10.6–14.7% were observed. The GA and the 1,5-AG/GA (AGI) ratio allow the characterization of T1D (child and adult-onset) and T2D with excellent sensitivity and specificity and can be recommended as tests that contribute to the diagnosis. The GA/HbA1c ratio adequately differentiates T1D in children but loses sensitivity for T1D in adults and T2D. For the diagnosis of GDM, the biomarkers under study showed a limited performance in how they were evaluated.

## Figures and Tables

**Table 1:** Characteristics of the main biomarkers to assess glycemic control.

Variables	Glycemia	HbA1c	1,5-AG	GA
Location	Serum/plasma	Erythrocytes	Serum/plasma	Serum/plasma
Conventional units	mg/dL	%	µg/mL	%
SI units	mmol/L	mmol/mol	µmol/L	mmol/mol
Duration of blood glucose reflected	8–10 h	2–3 months	1–2 weeks	2–3 weeks
Fasting required	Yes	No	No	No
Analytical variability, % (CV_A_) between run imprecision	2.8	1.2–1.5	2.1	1.6–2.8
Biological variability, % (CV_I_/CV_G_)	5.6/7.5	1.9/5.7	–	5.2/10.3
Advantages	Simple, reproducible, and low-cost quantification	Reflects long-term glycemic control	Assesses short-term blood glucose and postprandial fluctuations	Reflects short- to medium-term blood glucose control
	Criteria defined for diabetes and high-risk groups for diabetes development	Very stable, and little affected by diet or fasting		
		Gold standard for glycemic control	Associated with gestational diabetes and macrosomia	Not affected by the life span of erythrocytes, hemoglobinopathies, or autologous blood donation
		Well established in the literature for identifying risks of complications	Reliable marker of glycemic control in T2D with chronic kidney disease stage 1 to 3	More accurate assessment of recent glycemic control for treatment modifications in gestational and neonatal diabetes
Limitations	Requires fasting	Affected by red blood cell lifespan	Affected by diet, sex, race, and changes in renal threshold for glucose	Unreliable under conditions that alter the metabolism of serum proteins and albumin
	Affected by short-term lifestyle changes (stress, drugs, others)	Affected by hemoglobinopathies, iron deficiency, hemodialysis	Does not identify hypoglycemia	Cannot be used in patients with nephropathy and nephrotic syndrome
	Effect of “glycolysis” on the sample	Does not capture glycemic fluctuations in short periods	Limited in patients with chronic kidney disease stage 4 to 5, end-stage renal disease, and in those treated with SGLT–2i or acarbose	Affected by body fat content and thyroid hormones
	Inadequate to identify postprandial hyperglycemia	Higher cost in quantification		

1,5-AG: 1,5-anhydroglucitol; GA: glycated albumin; CV_A_: analytical coefficient of variation (analytical imprecision); CV_G_: inter-individual coefficient of variation; CV_I_: intra-individual coefficient of variation (within-subject); HbA1c: glycated hemoglobin fraction A1c; SI: International System of Units. References: [25,26,27,28,29,30,31,32,33].

**Table 2:** Anthropometric and laboratory characteristics of healthy groups.

Variable	Children	Adults	Pregnant Women **	*p*
n	299	290	406	
Age, y	10 (10–11)	43.5 (41–46)	26 (24–27)	<0.001
Sex, M/F	150/149	146/144	–	0.956 *
BMI, kg/m^2^	19.4 ± 4.1	27.3 ± 4.2	27.7 ± 5.0	<0.001
* Glycemia, mg/dL	90.0 (83–97)	91 (81–105)	69.0 (59–77)	<0.001
HbA1c, %	5.2 (5.1–5.4)	5.4 (5.1–5.6)	4.5 (4.4–4.7)	<0.001
1,5–AG, µg/mL	31.7 ± 8.6	28.9 ± 18.8	21.4 ± 7.7	<0.001
Urea, mg/dL	23.5 ± 6.0	26.0 ± 7.7	17.4 ± 4.6	<0.001
Creatinine, mg/dL	0.56 (0.46–0.64)	0.64 (0.52–0.76)	0.66 (0.60–0.72)	0.956
Hemoglobin, g/dL	13.4 ± 6.1	14.9 ± 6.7	12.3 ± 5.9	<0.001
Total protein, g/L	79 ± 8	69 ± 5	83 ± 7	<0.001
Albumin, gL	44 ± 3	39 ± 2	34 ± 3	<0.001
AST, U/L	24 (20–27)	16 (11–21)	15 (12–19)	<0.001
ALT, U/L	14 (10–17)	21 (17–26)	8 (6–12)	<0.001

n: sample size; 1,5-AG: 1,5-anhydroglucitol; ALT: alanine transaminase; AST: aspartate aminotransferase; BMI: body mass index; * Glycemia, non-fasting; M/F: male/female; ** 16–37 weeks of gestation. * Chi-squared for children vs. adults. Other comparisons were made by one-way analysis of variance or Kruskal–Wallis test.

**Table 3:** Reference interval for parametric glycated albumin.

Age	M/F	n	RI	90% Lower	90% Upper	Mean	Median
Children							
6–14 y	150/149	299	10.1–13.6	9.9–10.2	13.4–14.0	11.8	11.8
Adults							
21–62 y	146/144	290	9.9–13.9	9.8–10.1	13.8–14.2	11.9	11.9
Non-pregnant individuals							
6–62 y	296/293	589	10.0–13.8	9.5–10.1	13.7–13.9	11.9	11.9
Pregnant women **							
14–44 y	–	406	10.6–14.7	10.4–10.7	14.5–14.8	12.6	16.6

M/F: male/female; n: sample size; ** 16–37 weeks of gestation, RI: reference interval, calculated with a parametric method, and 90% lower and upper, 90% confidence interval. Children (normality K-S, *p* > 0.10), mean ± SD; 11.8 ± 0.89; adults (K-S, *p* > 0.10), mean ± SD, 11.9 ± 1.02; non-pregnant individuals, mean ± SD, 11.9 ± 0.96; pregnant women (K-S, *p* = 0.089), mean ± SD, 12.6 ± 1.05, glycated albumin measured with enzymatic method.

**Table 4:** Reference interval for 1,5-AG/glycated albumin ratio (AGI).

Age	M/F	N	RI	90% Lower	90% Upper	Mean	Median
Children							
6–14 y	150/149	299	1.2–4.3	0.9–1.4	4.2–4.8	2.45	2.59
Adults							
21–62 y	146/144	290	0.9–3.6	0.6–1.2	3.3–4.0	1.91	2.12
Non-pregnant individuals							
6–62 y	296/293	589	1.1–4.0	0.8–1.2	3.8–4.2	2.15	2.35
Pregnant women **							
14–44 y	–	406	0.8–3.1	0.7–0.9	3.9–3.3	1.56	1.67

M/F: male/female; n: sample size; ** weeks of gestation: 16–37, RI: reference interval, calculated with a non-parametric percentile method (2.5–97.5%), and 90% lower and upper, 90% confidence interval. 1,5-AG/GA ratio was calculated with biomarkers in µg/mL and %, respectively. Normality was rejected by Kolmogorov–Smirnov (*p* < 0.001) for all groups.

**Table 5:** Reference interval for glycated albumin/HbA1c ratio.

Age	M/F	N	RI	90% Lower	90% Upper	Mean	Median
Children							
6–14 y	150/149	299	1.8–2.6	1.8–1.9	2.2–2.7	2.27	2.26
Adults							
21–62 y	146/144	290	1.8–2.7	1.7–1.9	2.6–2.7	2.25	2.24
Non-pregnant individuals							
6–62 y	296/293	589	1.8–2.6	1.8–1.9	2.6–2.7	2.26	2.25
Pregnant women **							
14–44 y	–	406	2.3–3.6	2.2–2.3	3.3–4.1	2.81	2.77

M/F: male/female; n: sample size; ** weeks of gestation: 16–37, RI: reference interval, calculated with a non-parametric percentile method (2.5–97.5%), and 90% lower and upper, 90% confidence interval. GA/HbA1C ratio calculated with biomarkers in %. RI for children and adults with the parametric method (Kolmogorov–Smirnov, *p* > 0.10); RI for pregnant women with the non-parametric method (K-S; *p* < 0.05).

**Table 6:** Anthropometric and laboratory primary characteristics of the groups of people with diabetes.

Variable	T1D Children	T1D Adults	T2D	GDM
n	148	81	283	177
Age, y	11 (9–13)	44.5 (37–53)	60 (52–66)	31 (26–35)
Sex, M/F	72/76	34/47	79/204	–
BMI, kg/m^2^	18.8 ± 3.0	25.9 ± 4.7	29.8 ± 5.6	31.1 ± 5.2
Duration of diabetes, y	4.1 ± 3.1	15.2 ± 10.7	9.0 ± 6.1	–
Fasting glycemia, mg/dL	261 (171–349)	207.5 (137–271)	144.5 (107–190)	87 (79–95)
HbA1c, %	9.7 (8.7–11.0)	8.8 (7.7–9.9)	8.1 (6.7–9.4)	5.0 (4.8–5.3)

n: sample size; GDM: gestational diabetes mellitus, with 25.4 ± 3.7 weeks of gestation; T1D: type 1 diabetes; T2D: type 2 diabetes. The minimum and maximum concentrations for fasting blood glucose and HbA1c were 40–749 mg/dL and 5.6–19.4% for T1D children, 33–695 mg/dL and 4.7–17.7% for T1D adults, 37–511 mg/dL and 5.2—13.9% for T2D, and 53–125 mg/dL and 4.1–6.3% for GDM.

**Table 7:** Receiver operating characteristic curve parameters for glycated albumin.

Variable	T1D Children	T1D Adults	T2D	GDM
n, Disease/control	148/299	81/290	283/290	177/406
AUC, 95% CI	0.999 (0.99–1.00)	0.964 (0.94–0.98)	0.954	0.625 (0.58–0.66)
*p*	<0.0001	<0.0001	<0.0001	<0.0001
Youden index J	0.9899	0.9280	0.824	0.2436
Associated criterion, %	>14.3	>14.8	>14.2	>13.1
Sensitivity, %	99.3	93.8	84.8	53.7
Specificity, %	99.7	99.9	97.6	70.7

n: sample size; AUC: area under the curve; CI: confidence interval; T1D: type 1 diabetes; T2D: type 2 diabetes; GDM: gestational diabetes with 25.4 ± 3.7 weeks of gestation.

**Table 8:** Receiver operating characteristic curve parameters for the 1,5-AG/GA (AGI) ratio.

Variable	T1D Children	T1D Adults	T2D	GDM
n, Disease/control	148/299	81/290	283/290	177/406
AUC, 95% CI	1.000 (0.99–1.00)	0.993 (0.97–0.99)	0.993 (0.97–0.99)	0.705 (0.66–0.74)
*p*	<0.0001	<0.0001	<0.0001	<0.0001
Youden index J	0.993	0.993	0.939	0.351
Associated criterion, %	≤0.797	≤0.901	≤1.08	≤1.39
Sensitivity, %	99.3	97.4	96.3	63.1
Specificity, %	100.0	97.6	97.6	72.0

n: sample size; AUC: area under the curve; CI: confidence interval; T1D: type 1 diabetes; T2D: type 2 diabetes; GDM: gestational diabetes mellitus, with 25.4 ± 3.7 weeks of gestation. Ratio calculated with 1,5-anhydroglucitol in µg/mL and glycated albumin in %.

**Table 9:** Receiver operating characteristic curve parameters for the GA/HbA1c ratio.

Variable	T1D Children	T1D Adults	T2D	GDM
n, Disease/control	148/299	81/290	283/290	177/406
AUC, 95% CI	0.939 (0.91–0.96)	0.867 (0.83–0.90)	0.733 (0.69–0.77)	0.677 (0.64–0.71)
*p*	<0.0001	<0.0001	<0.0001	<0.0001
Youden index J	0.768	0.687	0.432	0.340
Associated criterion, %	>2.50	>2.56	>2.42	≤2.66
Sensitivity, %	87.2	76.2	61.2	62.2
Specificity, %	89.6	92.4	81.1	68.2

n: sample size; AUC: area under the curve; CI: confidence interval; T1D: type 1 diabetes; T2D: type 2 diabetes; GDM: gestational diabetes mellitus, with 25.4 ± 3.7 weeks of gestation. GA/HbA1c ratio calculated with biomarkers in %.

**Table 10:** Comparisons between the reference interval for glycated albumin in different populations measured by enzymatic methods.

Populations	Methods	n	Reference Interval, %	ΔRI	Reference
Euro-Brazilians Children and adults	Lucica GA-L	589	10.0–13.8	3.8	This study
^1^ Brazilians	GlycoGap	165	10.8–18.0	7.2	Chume et al. [49]
US Americans	Lucica GA-L	262	9.9–14.2	4.3	Tao et al. [50]
Hispanic or Latino individuals		24	10.1–14.2	4.1	
Non–Hispanic or non–Latino individuals		238	9.9–14.3	4.4	
Black US Americans		43	10.6–14.8	4.2	
Asians		41	10.6–14.8	4.2	
Caucasians		172	9.9–14.2	4.3	
US Americans	Lucica GA-L				Selvin et al. [51]
Total		1799	10.7–15.1	4.4	
Black individuals		261	10.9–15.5	4.6	
Caucasians		1538	10.7–14.9	4.2	
South Africans	QuantILab	663	10.7–15.2	4.5	Matsha et al. [48]
Italians	QuantILab	1334	Upper 12.0 Male	–	Bellia et al. [52]
			Upper 12.2 Female		
Italians	Lucica GA-L	32	11.7–16.9	5.2	Paroni et al. [29]
Europeans	QuantILab	252	9.0–16	7.0	Testa et al. [53]
Japanese	Norudia GA	1843	12.1–17.1	5.0	Koga et al. [54]
Japanese	Lucica GA-L	1575	12.2–16.5	4.3	Furusyo et al. [55]
^2^ Japanese blood donors	Lucica GA-L	3,142,794	10.4–17.2	6.8	Araki et al. [56]
Non-pregnant women	Lucica GA-L	32	12.0–16.2	4.2	Hiramatsu et al. [57]
^3^ Japanese neonates	Lucica GA-L	18	7.4–13.0	5.6	Suzuki et al. [58]
South Koreans	Norundia GA	120	11.2–17.5	6.3	Ha et al. [59]
Chinese	Lucica GA-L	458	20–59 y 10.4–13.9	3.5	Zhou et al. [60]
			60–79 y 10.2–14.8	4.6	
Total (min-max)	Enzymatic	–	9.0–18.0	3.8–7.2	–
**Pregnant women**					
Euro-Brazilians Pregnant women (16–37 weeks)	Lucica GA–L	406	10.6–14.7	4.1	This study
Caucasians	QuantLab	183	(1st T) 10.1–15.7	5.6	Agnello et al. [61]
			(2nd T) 10.5–15.5	5.0	
			(3rd T) 9.8–14.6	4.8	
Caucasians	Lucica GA-L	45	(6–10 w) 11.1–14.8	3.7	Paleari et al. [62]
			(16–18 w) 10.9–15.6	4.7	
			(24–28 w) 10.6–14.1	3.5	
			(>28 w) 10.7–14.3	3.6	
US Americans	Lucica GA-L	214	(10–14 w) 7.8–18.5	10.7	Pang et al. [63]
			(15–26 w) 8.5–38.4	29.9	
			(23–31 w) 8.7–19.8	11.1	
			(33–39 w) 7.7–18.0	10.3	
Japanese	Lucica GA-L	574	(1st T) 12.2–16.6	4.4	Hiramatsu et al. [57]
			(2nd T) 11.8–16.6	4.8	
			(3rd T) 11.3–15.3	4.0	
Chinese	GA Beijing	421	(1st T) 11.3–15.1	3.8	Dong et al. [64]
			(2nd T) 10.1–13.5	3.4	
			(3rd T) 9.8–13.1	3.3	
* Total (min-max)	Enzymatic	–	9.8–16.6	3.5–5.6	–

n: sample size; T: trimester; W: weeks of gestation; y: year; SD: standard deviation. ΔRI, variation of GA concentration from reference interval (difference highest-lowest). * Total (minimum–maximum) for pregnant women did not consider the study by Pang et al. [63]; GA Beijing, Glycated Albumin assay kit, Beijing Strong Biotechnologies Inc., Beijing, China; GlycoGap, Diazyme Laboratories, Poway, CA, USA; Lucica GA-L, Asahi Kasei Pharma Corporation, Tokyo, Japan; Norudia GA assay kits (Sekisui Medical Co., Ltd., Tokyo, Japan); QuantILab Glycated Albumin, Milan, Italy. ^1^ RI estimated as mean ± 2-SD of published data 14.4 ± 1.8 (mean ± 1-SD), ^2^ RI estimated as mean ± 2SD from blood donors (aged 16–60 years) of published data 13.8 ± 1.7% (mean ± 1-SD), ^3^ RI estimated as mean ± 2SD from neonate (aged 103 ± 60 days) of published data 10.2 ± 1.4% (mean ± 1-SD).

## Data Availability

The authors confirm that the data supporting the findings of this study are available within the article.

## Supplementary Materials

The following supporting information can be downloaded at: https://www.mdpi.com/article/10.3390/biomedicines12122651/s1, Table S1: Anthropometric and laboratory characteristics of the groups of people with diabetes; Table S2: Glycated albumin and comparison between groups and sexes; Table S3: 1,5-Anhydroglucitol and glycated albumin ratio and comparison between sexes; Table S4: 1,5-Anhydroglucitol and glycated albumin ratio and comparison between sexes.

## References

[1] IDF IDF DIABETES ATLAS. www.diabetesatlas.org.

[2] The Diabetes Control and Complications Trial Research Group (1993). The effect of intensive treatment of diabetes on the development and progression of long-term complications in insulin-dependent diabetes mellitus. New Engl. J. Med..

[3] The Diabetes Control and Complications Trial Research Group (1995). Effect of intensive therapy on the development and progression of diabetic nephropathy in the Diabetes Control and Complications Trial. Kidney Int..

[4] The Diabetes Control and Complications Trial Research Group (1995). The relationship of glycemic exposure (HbA1c) to the risk of development and progression of retinopathy in the diabetes control and complications trial. Diabetes.

[5] ElSayed N.A., Aleppo G., Bannuru R.R., Bruemmer D., Collins B.S., Ekhlaspour L., Gaglia J.L., Hilliard M.E., Johnson E.L., American Diabetes Association Professional Practice Committee (2024). 3. Prevention or Delay of Diabetes and Associated Comorbidities: Standards of Care in Diabetes-2024. Diabetes Care.

[6] Sato A., Yada S., Hosoba E., Kanno H., Miura H. (2019). Establishment of glycated albumin unit conversion equation from the standardized value (mmol/mol) to the routinely used value (%). Ann. Clin. Biochem..

[7] Sherwani S.I., Khan H.A., Ekhzaimy A., Masood A., Sakharkar M.K. (2016). Significance of HbA1c Test in Diagnosis and Prognosis of Diabetic Patients. Biomark. Insights.

[8] Beyond A1C Writing Group (2018). Need for Regulatory Change to Incorporate Beyond A1C Glycemic Metrics. Diabetes Care.

[9] Selvin E., Francis L.M., Ballantyne C.M., Hoogeveen R.C., Coresh J., Brancati F.L., Steffes M.W. (2011). Nontraditional markers of glycemia: Associations with microvascular conditions. Diabetes Care.

[10] Juraschek S.P., Steffes M.W., Selvin E. (2012). Associations of alternative markers of glycemia with hemoglobin A(1c) and fasting glucose. Clin. Chem..

[11] Liu L., Wan X., Liu J., Huang Z., Cao X., Li Y. (2012). Increased 1,5-anhydroglucitol predicts glycemic remission in patients with newly diagnosed type 2 diabetes treated with short-term intensive insulin therapy. Diabetes Technol. Ther..

[12] Zemlin A.E., Barkhuizen M., Kengne A.P., Erasmus R.T., Matsha T.E. (2019). Performance of glycated albumin for type 2 diabetes and prediabetes diagnosis in a South African population. Clin. Chim. Acta.

[13] Ying L., He X., Ma X., Shen Y., Su H., Peng J., Wang Y., Bao Y., Zhou J., Jia W. (2017). Serum 1,5-anhydroglucitol when used with fasting plasma glucose improves the efficiency of diabetes screening in a Chinese population. Sci. Rep..

[14] Dungan K.M., Buse J.B., Largay J., Kelly M.M., Button E.A., Kato S., Wittlin S. (2006). 1,5-anhydroglucitol and postprandial hyperglycemia as measured by continuous glucose monitoring system in moderately controlled patients with diabetes. Diabetes Care.

[15] Koga M. (2014). 1,5-Anhydroglucitol and glycated albumin in glycemia. Adv. Clin. Chem..

[16] Kim W.J., Park C.Y. (2013). 1,5-Anhydroglucitol in diabetes mellitus. Endocrine.

[17] Furusyo N., Hayashi J. (2013). Glycated albumin and diabetes mellitus. Biochim. Biophys. Acta.

[18] Koga M., Kasayama S. (2010). Clinical impact of glycated albumin as another glycemic control marker. Endocr. J..

[19] Rondeau P., Bourdon E. (2011). The glycation of albumin: Structural and functional impacts. Biochimie.

[20] Ciaccio M. (2019). Introduction of glycated albumin in clinical practice. J. Lab. Precis. Med..

[21] Qiu H.Y., Hou N.N., Shi J.F., Liu Y.P., Kan C.X., Han F., Sun X.D. (2021). Comprehensive overview of human serum albumin glycation in diabetes mellitus. World J. Diabetes.

[22] Tang M., Berg A.H., Zheng H., Rhee E.P., Allegretti A.S., Nigwekar S.U., Karumanchi S.A., Lash J.P., Kalim S. (2024). Glycated Albumin and Adverse Clinical Outcomes in Patients With CKD: A Prospective Cohort Study. Am. J. Kidney Dis. Off. J. Natl. Kidney Found..

[23] Wu W.C., Ma W.Y., Wei J.N., Yu T.Y., Lin M.S., Shih S.R., Hua C.H., Liao Y.J., Chuang L.M., Li H.Y. (2016). Serum Glycated Albumin to Guide the Diagnosis of Diabetes Mellitus. PLoS ONE.

[24] Inaba M., Okuno S., Kumeda Y., Yamada S., Imanishi Y., Tabata T., Okamura M., Okada S., Yamakawa T., Ishimura E. (2007). Glycated albumin is a better glycemic indicator than glycated hemoglobin values in hemodialysis patients with diabetes: Effect of anemia and erythropoietin injection. J. Am. Soc. Nephrol. JASN.

[25] Xu H., Pan J., Chen Q. (2024). The progress of clinical research on the detection of 1,5-anhydroglucitol in diabetes and its complications. Front. Endocrinol..

[26] Nathan D.M., Steffes M.W., Sun W., Rynders G.P., Lachin J.M. (2011). Determining stability of stored samples retrospectively: The validation of glycated albumin. Clin. Chem..

[27] Welter M., Boritza K.C., Anghebem-Oliveira M.I., Henneberg R., Hauser A.B., Rego F.G.M., Picheth G. (2018). Reference intervals for serum 1,5-anhydroglucitol in children, adolescents, adults, and pregnant women. Clin. Chim. Acta.

[28] Tseng K.B. (2023). Alternative Biomarkers for Assessing Glycemic Control for the Prognosis and Management of Diabetes. E-Da Med. J..

[29] Paroni R., Ceriotti F., Galanello R., Battista Leoni G., Panico A., Scurati E., Paleari R., Chemello L., Quaino V., Scaldaferri L. (2007). Performance characteristics and clinical utility of an enzymatic method for the measurement of glycated albumin in plasma. Clin. Biochem..

[30] Yazdanpanah S., Rabiee M., Tahriri M., Abdolrahim M., Rajab A., Jazayeri H.E., Tayebi L. (2017). Evaluation of glycated albumin (GA) and GA/HbA1c ratio for diagnosis of diabetes and glycemic control: A comprehensive review. Crit. Rev. Clin. Lab. Sci..

[31] Oğuz O., Mercan H., Hocaoglu-Emre F.S. (2021). Biological variation of glycated albumin, glucose and albumin in healthy Turkish subjects. Turk. J. Biochem..

[32] Montagnana M., Paleari R., Danese E., Salvagno G.L., Lippi G., Guidi G.C., Mosca A. (2013). Evaluation of biological variation of glycated albumin (GA) and fructosamine in healthy subjects. Clin. Chim. Acta.

[33] Kohzuma T., Yamamoto T., Uematsu Y., Shihabi Z.K., Freedman B.I. (2011). Basic Performance of an Enzymatic Method for Glycated Albumin and Reference Range Determination. J. Diabetes Sci. Technol..

[34] Ying L., Ma X., Shen Y., Lu J., Lu W., Zhu W., Wang Y., Bao Y., Zhou J. (2020). Serum 1,5-Anhydroglucitol to Glycated Albumin Ratio Can Help Early Distinguish Fulminant Type 1 Diabetes Mellitus from Newly Onset Type 1A Diabetes Mellitus. J. Diabetes Res..

[35] Sugawara D., Sato H., Makita E., Kuwata T., Takagi K., Ichihashi K. (2022). Clinical usefulness of glycated albumin and glycated albumin-to-glycated hemoglobin ratio of gestational diabetes mellitus in late pregnancy for predicting infant complications. Pediatr. Neonatol..

[36] Huh J.H., Kim K.J., Lee B.-W., Kim D.W., Kang E.S., Cha B.S., Lee H.C. (2014). The Relationship between BMI and Glycated Albumin to Glycated Hemoglobin (GA/A1c) Ratio According to Glucose Tolerance Status. PLoS ONE.

[37] Takeuchi T., Hirota Y., Nakagawa Y., Matsuoka A., Hamaguchi T., Okada Y., Sakaguchi K., Ogawa W., Koga M. (2022). Glycated albumin (GA) and the GA/HbA1c ratio are higher in diabetic patients positive for insulin antibodies with high binding capacity and low affinity. Diabetol. Int..

[38] Wang N., Xu Z., Han P., Li T. (2017). Glycated albumin and ratio of glycated albumin to glycated hemoglobin are good indicators of diabetic nephropathy in type 2 diabetes mellitus. Diabetes/Metab. Res. Rev..

[39] ElSayed N.A., Aleppo G., Bannuru R.R., Bruemmer D., Collins B.S., Ekhlaspour L., Gaglia J.L., Hilliard M.E., Johnson E.L., American Diabetes Association Professional Practice Committee (2024). 2. Diagnosis and Classification of Diabetes: Standards of Care in Diabetes-2024. Diabetes Care.

[40] Bell C.A. (1995). Clinical Guide to Laboratory Tests. 3rd edition. Norbert W. Tietz, ed. Transfusion.

[41] Hajian-Tilaki K. (2013). Receiver Operating Characteristic (ROC) Curve Analysis for Medical Diagnostic Test Evaluation. Casp. J. Intern. Med..

[42] Koga M., Hashimoto K., Murai J., Saito H., Mukai M., Ikegame K., Ogawa H., Kasayama S. (2011). Usefulness of glycated albumin as an indicator of glycemic control status in patients with hemolytic anemia. Clin. Chim. Acta.

[43] Tahara Y., Shima K. (1995). Kinetics of HbA1c, glycated albumin, and fructosamine and analysis of their weight functions against preceding plasma glucose level. Diabetes Care.

[44] Freitas P.A.C., Ehlert L.R., Camargo J.L. (2017). Glycated albumin: A potential biomarker in diabetes. Arch. Endocrinol. Metab..

[45] Kouzuma T., Uemastu Y., Usami T., Imamura S. (2004). Study of glycated amino acid elimination reaction for an improved enzymatic glycated albumin measurement method. Clin. Chim. Acta.

[46] Kouzuma T., Usami T., Yamakoshi M., Takahashi M., Imamura S. (2002). An enzymatic method for the measurement of glycated albumin in biological samples. Clin. Chim. Acta.

[47] Timbrell N.E. (2024). The Role and Limitations of the Reference Interval Within Clinical Chemistry and Its Reliability for Disease Detection. Br. J. Biomed. Sci..

[48] Matsha T.E., Korf M., Erasmus R.T., Hoffmann M., Mapfumo C., Smit F., Zemlin A.E. (2019). Reference interval determination for glycated albumin in defined subgroups of a South African population. Ann. Clin. Biochem..

[49] Chume F.C., Kieling M.H., Correa Freitas P.A., Cavagnolli G., Camargo J.L. (2019). Glycated albumin as a diagnostic tool in diabetes: An alternative or an additional test?. PLoS ONE.

[50] Tao X., Koguma R., Nagai Y., Kohzuma T. (2022). Analytical performances of a glycated albumin assay that is traceable to standard reference materials and reference range determination. J. Clin. Lab. Anal..

[51] Selvin E., Warren B., He X., Sacks D.B., Saenger A.K. (2018). Establishment of Community-Based Reference Intervals for Fructosamine, Glycated Albumin, and 1,5-Anhydroglucitol. Clin. Chem..

[52] Bellia C., Zaninotto M., Cosma C., Agnello L., Lo Sasso B., Bivona G., Plebani M., Ciaccio M. (2017). Definition of the upper reference limit of glycated albumin in blood donors from Italy. Clin. Chem. Lab. Med. CCLM/FESCC.

[53] Testa R., Ceriotti F., Guerra E., Bonfigli A.R., Boemi M., Cucchi M., Di Gaetano N., Santini G., Genovese S., Ceriello A. (2017). Glycated albumin: Correlation to HbA1c and preliminary reference interval evaluation. Clin. Chem. Lab. Med. CCLM/FESCC.

[54] Koga M., Shimizu I., Nakamura Y., Yamakado M. (2023). Establishment of a reference interval for glycated albumin based on medical check-up data from multiple medical institutions. Scand. J. Clin. Lab. Investig..

[55] Furusyo N., Koga T., Ai M., Otokozawa S., Kohzuma T., Ikezaki H., Schaefer E.J., Hayashi J. (2011). Utility of glycated albumin for the diagnosis of diabetes mellitus in a Japanese population study: Results from the Kyushu and Okinawa Population Study (KOPS). Diabetologia.

[56] Araki T., Ishikawa Y., Okazaki H., Tani Y., Toyooka S., Satake M., Miwa U., Tadokoro K. (2012). Introduction of glycated albumin measurement for all blood donors and the prevalence of a high glycated albumin level in Japan. J. Diabetes Investig..

[57] Hiramatsu Y., Shimizu I., Omori Y., Nakabayashi M. (2012). Determination of reference intervals of glycated albumin and hemoglobin A1c in healthy pregnant Japanese women and analysis of their time courses and influencing factors during pregnancy. Endocr. J..

[58] Suzuki S., Koga M., Takahashi H., Matsuo K., Tanahashi Y., Azuma H. (2012). Glycated albumin in patients with neonatal diabetes mellitus is apparently low in relation to glycemia compared with that in patients with type 1 diabetes mellitus. Horm. Res. Paediatr..

[59] Ha C., Oh J., Park H.-D. (2021). Evaluation of the Analytical Performance of the Norudia GA Glycoalbumin Test. Lab. Med. Online.

[60] Zhou Q., Shi D.B., Lv L.Y. (2017). The establishment of biological reference intervals of nontraditional glycemic markers in a Chinese population. J. Clin. Lab. Anal..

[61] Agnello L., Lo Sasso B., Scazzone C., Giglio R.V., Gambino C.M., Bivona G., Pantuso M., Ciaccio A.M., Venezia R., Vidali M. (2021). Preliminary reference intervals of Glycated Albumin in healthy Caucasian pregnant women. Clin. Chim. Acta.

[62] Paleari R., Vidali M., Ceriotti F., Pintaudi B., Luisa De Angelis M., Vitacolonna E., Cataldo I., Torlone E., Succurro E., Angotti E. (2023). Reference intervals for glycated albumin during physiological pregnancy of Europid women: Evidences from a prospective observational study. Clin. Chim. Acta.

[63] Pang W.W., Hinkle S.N., Wu J., Stallcup P., Tsai M.Y., Sacks D.B., Zhang C. (2023). A Longitudinal Study of Plasma Glycated Albumin across Pregnancy and Associations with Maternal Characteristics and Cardiometabolic Biomarkers. Clin. Chem..

[64] Dong Y., Zhai Y., Wang J., Chen Y., Xie X., Zhang C., Liu J., Lu Y., Tang G., Han L. (2020). Glycated albumin in pregnancy: Reference intervals establishment and its predictive value in adverse pregnancy outcomes. BMC Pregnancy Childbirth.

[65] Sako T., Mori A., Lee P., Takahashi T., Izawa T., Karasawa S., Furuuchi M., Azakami D., Mizukoshi M., Mizutani H. (2008). Diagnostic Significance of Serum Glycated Albumin in Diabetic Dogs. J. Vet. Diagn. Investig..

[66] Sugawara D., Sato H., Ichihashi K., Nagai K., Kawano A. (2018). Glycated albumin level during late pregnancy as a predictive factor for neonatal outcomes of women with diabetes. J. Matern.-Fetal Neonatal Med..

[67] Shimizu I., Kohzuma T., Koga M. (2019). A proposed glycemic control marker for the future: Glycated albumin. J. Lab. Precis. Med..

[68] Chume F.C., Freitas P.A.C., Schiavenin L.G., Pimentel A.L., Camargo J.L. (2022). Glycated albumin in diabetes mellitus: A meta-analysis of diagnostic test accuracy. Clin. Chem. Lab. Med. CCLM/FESCC.

[69] Chume F.C., Freitas P.A.C., Schiavenin L.G., Sgarioni E., Leitao C.B., Camargo J.L. (2024). Glycated albumin in the detection of diabetes during COVID-19 hospitalization. PLoS ONE.

[70] Dozio E., Di Gaetano N., Findeisen P., Corsi Romanelli M.M. (2017). Glycated albumin: From biochemistry and laboratory medicine to clinical practice. Endocrine.

[71] Sugawara D., Makita E., Matsuura M., Sato H., Kuwata T., Ichihashi K. (2023). Prepregnancy body mass index and glycated albumin in the third trimester may predict infant complications in gestational diabetes mellitus: A retrospective cohort study. Diabetol. Int..

[72] Song S.O., Yun J.S., Ko S.H., Ahn Y.B., Kim B.Y., Kim C.H., Jeon J.Y., Kim D.J., Seo D.H., Kim S.H. (2022). Prevalence and clinical characteristics of fulminant type 1 diabetes mellitus in Korean adults: A multi-institutional joint research. J. Diabetes Investig..

[73] Imagawa A., Hanafusa T., Uchigata Y., Kanatsuka A., Kawasaki E., Kobayashi T., Shimada A., Shimizu I., Toyoda T., Maruyama T. (2003). Fulminant type 1 diabetes: A nationwide survey in Japan. Diabetes Care.

[74] Hwang Y.-C., Jung C.H., Ahn H.-Y., Jeon W.S., Jin S.-M., Woo J.-t., Cha B.S., Kim J.H., Park C.-Y., Lee B.-W. (2014). Optimal glycated albumin cutoff value to diagnose diabetes in Korean adults: A retrospective study based on the oral glucose tolerance test. Clin. Chim. Acta.

[75] Akatsuka J., Mochizuki M., Musha I., Ohtake A., Kobayashi K., Kikuchi T., Kikuchi N., Kawamura T., Urakami T., Sugihara S. (2015). The ratio of glycated albumin to hemoglobin A1c measured in IFCC units accurately represents the glycation gap. Endocr. J..

[76] Kim D., Kim K.J., Huh J.H., Lee B.W., Kang E.S., Cha B.S., Lee H.C. (2012). The ratio of glycated albumin to glycated haemoglobin correlates with insulin secretory function. Clin. Endocrinol..

[77] Koga M. (2014). Glycated albumin; clinical usefulness. Clin. Chim. Acta.

[78] Aihara M., Jinnouchi H., Yoshida A., Ijima H., Sakurai Y., Hayashi T., Koizumi C., Kubota T., Usami S., Yamauchi T. (2023). Evaluation of glycated albumin levels in tears and saliva as a marker in patients with diabetes mellitus. Diabetes Res. Clin. Pract..

