# Correction: Al-Lahham et al. Reference Interval for Glycated Albumin, 1,5-AG/GA, and GA/HbA1c Ratios and Cut-Off Values for Type 1, Type 2, and Gestational Diabetes: A Cross-Sectional Study. *Biomedicines* 2024, *12*, 2651

**DOI:** 10.3390/biomedicines13071621

**Published:** 2025-07-02

**Authors:** Yusra Al-Lahham, Waldemar Volanski, Liana Signorini, Ademir Luiz do Prado, Glaucio Valdameri, Vivian Rotuno Moure, Marciane Welter, Alexessander C. Alves, Marcel Henrique Marcondes Sari, Fabiane Gomes de Moraes Rego, Geraldo Picheth

**Affiliations:** 1 Graduate Program in Pharmaceutical Sciences, Federal University of Paraná, Curitiba 80210-170, Brazil; yusra.lahham@gmail.com (Y.A.-L.); volanski@ufpr.br (W.V.); lianasig@ufpr.br (L.S.); ademir.prado@ifpr.edu.br (A.L.d.P.); gvaldameri@ufpr.br (G.V.); vivian.moure@ufpr.br (V.R.M.); marcianewelter@yahoo.com.br (M.W.); marcelsari@ufpr.br (M.H.M.S.); rego@ufpr.br (F.G.d.M.R.); 2 Laboratory Division, Curitiba City Hall, Curitiba 80530-908, Brazil; 3 Federal Institute of Parana, Colombo 83403-515, Brazil; 4 School of Human Development and Health, Faculty of Medicine, University of Southampton, Southampton SO17 1BJ, UK; a.couto-alves@soton.ac.uk

## 1. Error in Table

In the original publication [1], there was a mistake in Table 4. Reference interval for 1,5-AG/glycated albumin ratio (AGI), as published. The mistake which was made was in the pregnant women values. The corrected Table 4 appears below. The authors state that the scientific conclusions are unaffected. This correction was approved by the Academic Editor. The original publication has also been updated.

## 2. Error in Table S3

In the original publication, there was a mistake in Table S3. 1,5-Anhydroglucitol and glycated albumin ratio and comparison between sexes, as published. The mistake which was made was in the 1,5-AG/GA mean ± SD values. The corrected Table S3 appears below. The authors state that the scientific conclusions are unaffected.

## 3. Table Title

In the original publication, there was a mistake in the title for Table S4. 1,5-Anhydroglucitol and glycated albumin ratio and comparison between sexes. The correct title appears below. All values in the original Table S4 are correct. The authors state that the scientific conclusions are unaffected.

## Figures and Tables

**Table 4:** Reference interval for 1,5-AG/glycated albumin ratio (AGI).

Age	M/F	n	RI	90% Lower	90% Upper	Mean	Median
Children							
6–14 y	150/149	299	1.2–4.3	0.9–1.4	4.2–4.8	2.45	2.59
Adults							
21–62 y	146/144	290	0.9–3.6	0.6–1.2	3.3–4.0	1.91	2.12
Non-pregnant individuals							
6–62 y	296/293	589	1.1–4.0	0.8–1.2	3.8–4.2	2.15	2.35
Pregnant women **							
14–44 y	–	406	0.8–3.1	0.7–0.9	2.9–3.3	1.56	1.67

M/F: male/female; n: sample size; ** weeks of gestation: 16–37, RI: reference interval, calculated with a non-parametric percentile method (2.5–97.5%), and 90% lower and upper, 90% confidence interval. 1,5-AG/GA ratio was calculated with biomarkers in µg/mL and %, respectively. Normality was rejected by Kolmogorov–Smirnov (*p* < 0.001) for all groups.

**Table S3:** 1,5-Anhydroglucitol and glycated albumin ratio and comparison between sexes.

Group	Sex	n	1,5-AG/GA Mean ± SD	*p*
Children (control)	Male	150	2.81 ± 0.81	**0.004**
	Female	149	2.56 ± 0.68	
T1D children	Male	72	0.17 ± 0.17	0.083
	Female	76	0.12 ± 0.11	
Adults (control)	Male	146	2.92 ± 2.63	**0.001**
	Female	144	2.08 ± 1.38	
T1D adults	Male	34	0.21 ± 0.71	0.788
	Female	47	0.17 ± 0.62	
T2D adults	Male	79	0.23 ± 0.19	0.487
	Female	204	0.25 ± 0.25	

^1^n: sample size; T1D: type 1 diabetes; T2D: type 2 diabetes. 1,5-Anhydroglucitol (µg/mL) and glycated albumin (%) ratio. Comparisons were made by two-tailed Student’s *t*-test. Significant values (*p* < 0.05) are marked in bold.

**Table S4:** Glycated albumin and HbA1c ratio and comparison between sexes.

Group	Sex	n	GA/HbA1c Mean ± SD	*p*
Children (control)	Male	150	2.32 ± 0.41	0.072
	Female	149	2.23 ± 0.45	
T1D children	Male	72	2.97 ± 0.46	0.309
	Female	76	2.90 ± 0.32	
Adults (control)	Male	146	2.21 ± 0.99	0.202
	Female	144	2.39 ± 1.38	
T1D adults	Male	34	3.04 ± 1.15	0.457
	Female	47	2.86 ± 1.01	
T2D adults	Male	79	2.65 ± 0.50	0.157
	Female	204	2.55 ± 0.53	

^1^n: sample size; T1D: type 1 diabetes; T2D: type 2 diabetes. Glycated albumin (%) and HbA1c (%) ratio. Comparisons were made by two-tailed Student’s *t*-test. Significant values are presented as *p* < 0.05.
