# Supplementary material for: Reference Interval for Glycated Albumin, 1,5-AG/GA, and GA/HbA1c Ratios and Cut-Off Values for Type 1, Type 2, and Gestational Diabetes: A Cross-Sectional Study
Source: Biomedicines. 2024 Nov 21;12(12):2651. doi: 10.3390/biomedicines12122651 (PMC11673511; doi:10.3390/biomedicines12122651)
Supplement: Supplementary file 1 [file biomedicines-12-02651-s001.zip › biomedicines-3314928-supplementary material.pdf]

biomedicines

MDPI

Supplementary Material

Table S1. Anthropometric and laboratory characteristics of the diabetic groups.

Variable	T1D Children	T1D Adults	T2D	GDM
n	148	81	283	177
Age, y	11 (9–13)	44.5 (37–53)	60 (52–66)	31 (26–35)
Sex, M/F	72/76	34/47	79/204	–
BMI, kg/m²	18.8 ± 3.0	25.9 ± 4.7	29.8 ± 5.6	31.3 ± 5.2
Duration of diabetes, y	4.1 ± 3.1	15.2 ± 10.7	9.0 ± 6.1	–
Fasting glycemia, mg/dL	261 (171–349)	207.5 (137–271)	144.5 (107–190)	87 (79–95)
HbA1c, %	9.7 (8.7–11.0)	8.8 (7.7–9.9)	8.1 (6.7–9.4)	5.0 (4.8–5.3)
1,5-AG, µg/mL	3.7 ± 2.9	5.5 ± 6.0	5.8 ± 6.7	18.0 ± 8.2
Glycated albumin, %	29.6 ± 7.6	25.6 ± 6.5	21.3 ± 7.3	13.2 ± 1.5
1,5-AG/GA ratio	0.15 ± 0.14	0.24 ± 0.31	0.28 ± 0.29	1.36 ± 0.66
GA/HbA1c ratio	2.93 ± 0.39	2.87 ± 0.56	2.58 ± 0.52	1.36 ± 0.28
Creatinine, mg/dL	0.70 (0.62–0.80)	0.90 (0.80–1.1)	0.90 (0.80–1,0)	0.66 (0.60–0.72)
Total protein, g/L	73 ± 6	83 ± 9	87 ± 6	83 ± 7
Albumin, gL	43 ± 4	41 ± 4	41 ± 4	34 ± 3
AST, U/L	21 (18–25)	15.5 (12–21.5)	20 (16–25)	–
ALT, U/L	13 (10–16)	17 (9–22)	20 (16–25)	–

[1]The values are mean ± standard deviation, median (25%–75%) or n. n: sample size; GDM: gestational diabetes mellitus, with 25.4 ± 3.7 weeks of gestation; 1,5-AG: 1,5-anhydroglucitol; ALT: alanine transaminase; AST: aspartate aminotransferase; BMI: body mass index; M/F: male/female. The 1,5-AG/GA ratio was calculated with 1,5-anhydroglucitol in µg/mL and glycated albumin in %. The GA/HbA1c ratio was obtained with GA in % and HbA1c in %.

Table S2. Glycated albumin and comparison between groups and sexes.

Group	Sex	n	GA¸% mean ± SD	*p*
Children (control)	Male	150	12.1 ± 0.96	0.066
	Female	149	11.9 ± 0.91	
T1D children	Male	72	30.6 ± 9.1	0.498
	Female	76	29.2 ± 5.9	
Adults (control)	Male	146	11.9 ± 1.5	0.596
	Female	144	12.0 ± 1.7	
T1D adults	Male	34	24.6 ± 7.7	0.440
	Female	47	25.8 ± 6.0	
T2D adults	Male	79	22.4 ± 7.3	0.343
	Female	204	21.5 ± 7.1	

[1]n: sample size; GA: glycated albumin; T1D: type 1 diabetes; T2D: type 2 diabetes. Comparisons were made by two-tailed Student's t-test. Significant values are presented as $p < 0.05$.

Table S3. 1,5-Anhydroglucitol and glycated albumin ratio and comparison between sexes.

Group	Sex	n	1,5-AG/GA mean ± SD	*p*
Children (control)	Male	150	2.81 ± 0.81	**0.004**
	Female	149	2.56 ± 0.68	
T1D children	Male	72	0.17 ± 0.17	0.083
	Female	76	0.12 ± 0.11	
Adults (control)	Male	146	2.92 ± 2.63	**0.001**
	Female	144	2.08 ± 1.38	
T1D adults	Male	34	0.21 ± 0.71	0.788
	Female	47	0.17 ± 0.62	
T2D adults	Male	79	0.23 ± 0.19	0.487
	Female	204	0.25 ± 0.25	

[1]n: sample size; T1D: type 1 diabetes; T2D: type 2 diabetes. 1,5-Anhydroglucitol (μg/mL) and glycated albumin (%) ratio. Comparisons were made by two-tailed Student's t-test. Significant values ($p < 0.05$) are marked in bold.

Table S4. Glycated albumin and HbA1c ratio and comparison between sexes.

Group	Sex	n	GA/HbA1c mean ± SD	*p*
Children (control)	Male	150	2.32 ± 0.41	0.072
	Female	149	2.23 ± 0.45	
T1D children	Male	72	2.97 ± 0.46	0.309
	Female	76	2.90 ± 0.32	
Adults (control)	Male	146	2.21 ± 0.99	0.202
	Female	144	2.39 ± 1.38	
T1D adults	Male	34	3.04 ± 1.15	0.457
	Female	47	2.86 ± 1.01	
T2D adults	Male	79	2.65 ± 0.50	0.157
	Female	204	2.55 ± 0.53	

[1]n: sample size; T1D: type 1 diabetes; T2D: type 2 diabetes. Glycated albumin (%) and HbA1c (%) ratio. Comparisons were made by two-tailed Student's t-test. Significant values are presented as $p < 0.05$.
